# Factors affecting adherence to national malaria treatment guidelines in management of malaria among public healthcare workers in Kamuli District, Uganda

**DOI:** 10.1186/s12936-016-1153-5

**Published:** 2016-02-24

**Authors:** Charles Bawate, Sylvia T. Callender-Carter, Ben Nsajju, Denis Bwayo

**Affiliations:** Kamuli District Local Government, Kamuli, Uganda; BLC Research Centre, Iganga, Uganda; Civil Society Fund, Kampala, Uganda; Department of Public Health, Bugema University, Kampala, Uganda

**Keywords:** Adherence, National malaria treatment guidelines, Uganda

## Abstract

**Background:**

Malaria remains a major public health threat accounting for 30.4 % of disease morbidity in outpatient clinic visits across all age groups in Uganda. Consequently, malaria control remains a major public health priority in endemic countries such as Uganda. Experiences from other countries in Africa that revised their malaria case management suggest that health workers adherence may be problematic.

**Methods:**

A descriptive, cross-sectional design was used and collected information on health system, health workers and patients. Using log-binomial regression model, adjusted prevalence risk ratios (PRRs) and their associated 95 % confidence intervals were determined in line with adherence to new treatment guidelines of parasitological diagnosis and prompt treatment with artemisinin combination therapy (ACT).

**Results:**

Nine health centres, 24 health workers and 240 patient consultations were evaluated. Overall adherence to national malaria treatment guidelines (NMTG) was 50.6 % (122/241). It was significantly high at HC III [115 (53 %)] than at HC IV (29 %) [PRR = 0.28 (95 % CI 0.148 0.52), p = 0.000]. Compared to the nursing aide, the adherence level was 1.57 times higher among enrolled nurses (p = 0.004) and 1.68 times higher among nursing officers, p = 0.238, with statistical significance among the former. No attendance of facility malaria-specific continuing medical education (CME) sessions [PRR = 1.9 (95 % CI 1.29 2.78), p = 0.001] and no display of malaria treatment job aides in consultation rooms [PRR = 0.64 (95 % CI 0.4 1.03), p = 0.07] was associated with increased adherence to guidelines with the former showing a statistical significance and the association of the latter borderline statistical significance. The adherence was higher when the laboratory was functional [PRR = 0.47 (95 % CI 0.35 0.63)] when the laboratory was functional in previous 6 months. Age of health worker, duration of employment, supervision, educational level, and age of patient were found not associated with adherence to new treatment guidelines.

**Conclusion:**

Adherence to malaria treatment guidelines in Uganda is sub-optimal. There is an urgent need for deliberate interventions to improve adherence to these guidelines. Possible interventions to be explored should include: provision of job aides and improved access to laboratory services. There is also a need for continuous medical educational sessions for health workers, especially those at higher-level facilities and higher cadres, on adherence to guidelines in management of fever, including management of other causes of fever.

## Background

The World Health Organization (WHO) estimated that there were 214 million cases of malaria in 2015 and 438,000 deaths mostly young children in sub-Saharan Africa [1]. On the African continent, at least 12 billion US dollars are spent in malaria costs of illness, treatment and premature death [2]. A prompt and accurate diagnosis of malaria is the key to effective management of malaria [3–6]. Effective case management of malaria is a major strategy that has been adopted by several countries. It entails proper clinical assessment, laboratory confirmation of the disease either by light microscopy or rapid diagnostic test (RDT) prior to treatment with an effective anti-malarial [1, 4]. In Uganda, malaria remains the leading cause of morbidity (30.4 %) and mortality (12.8 %) in its hospitals [7], with an estimated 12 million clinical cases treated annually in the public health system alone [3]. Clinical diagnosis, using fever as the major symptom, is the most widely used approach by practitioners at all levels of training. This approach has been practice in Uganda for a long time [8]. Moreover, a large number of outpatients do not have malaria parasitaemia. This creates a diagnostic dilemma and reduces the accuracy of this criterion for diagnosis of malaria [5]. The Uganda Ministry of Health (MOH) recommends the WHO parasitological confirmation of malaria either by microscopy or RDT before initiation of treatment as per the national malaria treatment guidelines (NMTG) [5, 6].

Of all malaria-endemic countries in Africa, Uganda ranked third with an average of 30 % of all outpatient department (OPD) clinic visits due to malaria (with most diagnoses made clinically) and with between 20 and 50 % of all hospital admissions a consequence of malaria [9].These admissions are due to late presentation, inadequate management and unavailability or stock-outs of effective drugs [8]. This high burden may be partly a result of misdiagnosis, since many facilities lack laboratory capacity and it is often difficult to clinically distinguish malaria from other infectious diseases. However, malaria remains responsible for a high proportion of public health expenditure on curative treatment, and efforts to reduce malaria incidence would free up available health resources and facilities and health workers’ time to tackle other health issues. Experiences from other countries in sub-Saharan Africa that revised their malaria case management to artemisinin combination therapy (ACT) suggest that health workers’ adherence may be problematic [10–14]. In Kenya, 3 years after the implementation of a protocol stipulating routine malaria microscopy or RDT testing of all adults presenting with fever and prescription of artemether/lumefantrine (AL) to only those who test positive, the testing rates in health facilities with diagnostic malaria services available did not exceed 54 % and nearly a third of test negative cases were prescribed AL [15]. Although a Ugandan study showed use of ACT prevailing over non-recommended therapy, the quality of case management of malaria was not optimal [15]. However, in another review it was noted that health workers’ adherence to revised anti-malarial prescription protocols generally improves over time [16]. Low laboratory staff numbers, non-use of standard treatment guidelines, delay in producing laboratory results, and belief that patients with negative malaria results improved upon treatment with anti-malarials, were some of the factors responsible for low adherence to laboratory results [17, 18].

In Uganda, clinical diagnosis using fever as the major symptom is the most widely used approach by practitioners at all levels of training. Despite fever being a major symptom of malaria, it can occur in other illnesses as well. This creates a diagnostic dilemma and reduces the accuracy of this criterion for diagnosis of malaria [3, 5]. Effective translation of new guidelines into clinical practice is of critical importance to maximize the potential of improved, efficacious therapy and curtail the cost of malaria health care. Despite changes in policy recommendations and reported benefits, reports indicate varying levels of healthcare workers’ adherence to new malaria management guidelines [10–19]. This study was conducted to determine levels of adherence to the national guidelines in the management of uncomplicated malaria in a rural district in Uganda and explore possible reasons for health workers’ adherence or non-adherence.

## Methods

### Definitions

The study definitions reflected national malaria case management guidelines from the latest Uganda National Malaria Control Programme [3, 6]. In summary, the guidelines recommend parasitological confirmation of malaria either by microscopy or malaria RDT before initiation of treatment among patients presenting with fever or history of fever across all age groups. Adherence to guidelines meant that a patient suspected of malaria had: (i) a blood smear or malaria RDT done; (ii) if tested positive, s/he was prescribed only a recommended anti-malarial; and, (iii) if negative, s/he was not given any anti-malarial at all. Non-adherence to guidelines meant that a patient suspected of malaria had: (i) a blood test to confirm malaria was not done; (ii) a blood test was negative but s/he was still prescribed an anti-malarial; and, (iii) a blood test was positive but no anti-malarial or an incorrect anti-malarial, such as chloroquine was prescribed. A health centre (HC) IV or health sub-district is the level of service delivery at county level serving a population of 100,000 people. It offers preventive, curative and rehabilitative care in its catchment areas. It is the second level referral service for life-saving medical, surgical and obstetric care and provision of the physical base of the health sub-districts (HSD) management team. Health centre III offers services at sub-county level for a population of about 20,000, and includes continuous preventive, promotive and curative care services and supervises HC II facilities under its jurisdiction [20].

### Study area and design

A descriptive, cross-sectional research design was employed during December 2014. Pre-testing of the semi-structured questionnaire was done outside the facilities studied. Cronbach alpha coefficient was used to ensure internal consistency of the research tool. Individual patient records and prescription were collected and results were linked to the healthcare provider through special identifier codes. Healthcare workers who were attending to inpatients and maternity clinics were not involved in this study. Patients who had come for follow-up visits for chronic conditions such as tuberculosis and diabetes, traumas, burns, and patients referred or admitted for hospitalization were not part of the study. Collected data included demographics, presenting complaint, presumptive diagnosis, laboratory test results, drugs prescribed, and presence of job aides. Other facility and health worker-related data captured were presence of anti-malarial drugs (ACT) in facility stores, supervision reports, malaria-specific refresher training attended, and satisfaction level of patients. Nine public health facilities (eight HCIIIs and one HC IV) in Kamuli District, Uganda were studied. The names of all HC III and IV public facilities were written on small pieces of paper which were then folded and put in an empty box. Nine facilities were then drawn out of the possible 12 by simple random sampling. Each time a facility was picked, the paper was not replaced. The facilities were drawn from three HSDs, namely Buzaaya, Bugabula North and Bugabula South in Kamuli. The facilities were selected based on the following criteria: (i) availability of laboratory facility for malaria diagnosis (malaria microscopy or RDT performance); and, (ii) had to be level III or IV.

At the facility, three health workers were randomly selected among those found on duty. Twenty-seven patients were systematically sampled out at outpatient department at exit per facility. Observations were conducted at exit to minimize Hawthorne effect. A first case was identified at exit and then every third person who met the criteria was enrolled in the study. In case anyone declined, the immediate next person would be approached. The process continued until the number of 27 patients was reached. These patients were identified within 1–2 days depending on how busy the facilities were.

Kamuli District is located in southeastern Uganda. The climate is typically tropical with two distinct seasons.

### Data management and statistical analyses

Completed questionnaires once reviewed at the end of the day were filed for data entry. Data entry was designed in Epi-data version 3.1 with in-built checks and double-entry to minimize data-entry errors. Data were entered and secured on a password-protected computer. Data were transferred from Epi-data to Stata^®^ version 11(StataCorp, College Station, TX, USA) software for statistical analyses. Exploratory analyses were done to check for cleanliness and any outliers, or erroneous-looking data were cross-checked and cleaned where errors were identified.

Prevalence risk ratios (PRRs) were used as a measure of association because the outcome (adherence to guidelines) was high [16]. For multivariate analysis binomial regression models were used to determine the adjusted PRRs and their associated 95 % confidence intervals. Multivariable models were adjusted for potential confounders, which were added if they showed statistical significance of 0.10 or less at bivariate analysis, and other factors were added to the model if they had been found to be significantly associated with adherence to these guidelines in related studies.

### Ethical approval

Ethical approval for this study was received from the Bugema University School of Graduate Studies Ethical Review Board and Kamuli District local government also provided clearance.

## Results

### Characteristics of study respondents

Nine health centres were visited during the study time (eight HC III and one HC IV). The mean age and median for health workers were 33.32 and 34 years, respectively. There were an equal number (12) of health workers by gender. Majority of the health workers had formal health-related education level with only two (8.3 %) not having formal medical training. A total of 240 patient records were reviewed at exit with 88 (36.7 %) of the patients being children under 5 years of age and 159 (66.25 %) were female (Table 1).

**Table 1:** Characteristics of study respondents

Variable	Total N (%)	Facility level	
		8 HC III N (%)	1 HC IV N (%)
Healthcare workers			
Age			
Mean (SD)	33.32 (7.97)		
Median (Range)	34 (30)		
Gender of respondents			
Male	12	11 (91.67)	1 (8.33)
Female	12	10 (83.33)	2 (16.67)
Educational level			
No formal education	2	2 (100.0)	0 (0.0)
Certificate holder	13	11 (84.62)	2 (15.38)
Diploma holder	9	8 (88.89)	1 (11.11)
Patients			
Age			
Under 5 years	88	85 (96.59)	3 (3.41)
Over 5 years	152	130 (85.53)	22 (14.47)
Gender			
Male	81	78 (96.3)	3 (3.7)
Female	159	137 (86.16)	22 (13.84)

### Adherence to national malaria treatment guidelines

The overall prevalence of adherence to guidelines was 50.6 % (122/241). The adherence to guidelines was 76.14 % among those who were tested and found negative. Only 42.97 % of those who tested positive were managed according to policy guidelines. All patients who did not access laboratory services were managed contrary to new policy guidelines (Table 2).

**Table 2:** Adherence to national malaria treatment guidelines

Variable	Adhered to guidelines N (%)	Did not adhere to guidelines N (%)	P value
Adherence level			
All	122 (50.62)	119 (49.38)	
Facility level			
HC III	115 (53.0)	102 (47.0)	
HC IV	7 (29.17)	17 (70.83)	0.027
Laboratory findings			
Malaria positive	55 (42.97)	73 (57.03)	
Malaria negative	67 (76.14)	21 (23.86)	
No laboratory findings	0 (0)	25 (100)	

Adherence to NMTG was significantly higher at HC III [115 (53 %)] than at HC IV (29 %) [adjusted PRR = 0.28 (95 % CI 0.148 0.52), p = 0.000]. Compared to the nursing aides, the adherence level was 1.57 times higher among enrolled nurses (p = 0.004) and 1.68 times higher among nursing officers, p = 0.238 with statistical significance among the former. Non-attendance of facility continuing medical education (CME) session on malaria [adjusted PRR = 1.9 (95 % CI 1.29 2.78), p = 0.001] and no display of malaria treatment job aides in consultation rooms [adjusted PRR = 0.64 (95 % CI 0.4 1.03), p = 0.07] were associated with increased adherence to guidelines with the former showing a statistical significance and the association of the latter borderline statistical significance. Presence of a functional laboratory was found to be associated with adherence to the guidelines. The adjusted PRRs were 0.47 (95 % CI 0.35 0.63) when the laboratory was functional in previous 6 months. The following factors were found not to be associated with adherence to guidelines in this study: age of health worker, educational level of health worker and age of patient (Table 3).

**Table 3:** Prevalence, unadjusted and adjusted prevalence risk ratios (PRRs) of factors associated with adherence to national malaria treatment guidelines among health workers in Kamuli District, Uganda

Characteristic	Adhered N (%)	Unadjusted PRRs (95 % CI)	P value	Adjusted PRRs (95 % CI)	P value
All	122/241 (50.6)				
Age of health worker (years)					
21–30	60 (53.6)	1		–	
31–40	40 (46.5)	1.04 (0.74 1.47)	0.79	–	
>40	22 (51.6)	0.91 (0.63 1.32)	0.61	–	
Facility level					
HC III	115 (53)	1		1	
HC IV	7 (29)	0.55 (0.29 1.04)	0.06	0.28 (0.148 0.52)	0.000
Gender of health worker					
Male	49 (40.1)	1		–	
Female	73 (59.8)	0.87 (0.68 1.12)	0.29	–	
Highest educational level					
Below certificate	11 (55)	1		–	
Certificate	56 (57.1)	1.03 (0.67 1.6)	0.86	–	
Diploma	55 (45.1)	0.82 (0.53 1.27)	0.38	–	
Category of health worker					
Nurse aide	11 (55)	1		1	
Enrolled nurse	50 (55.6)	0.91 (0.64 1.29)	0.62	1.57 (1.4 1.75)	0.004
Nursing officer	13 (52.0)	0.85 (0.53 1.38)	0.53	1.68 (1.35 2.20)	0.238
Clinical officer	42 (42.9)	0.71 (0.48 1.02)	0.38	1.16 (0.76 1.76)	0.48
Refresher training in last 2 years					
Yes	76 (62.3)	1		–	
No	46 (37.7)	1.07 (0.83 1.38)	0.59	–	
Attended facility CME on malaria in last year					
Yes	105 (49.3)	1		1	
No	5 (25.0)	1.52 (1.14 2.02)	0.004	1.9 (1.29 2.78)	0.001
Malaria treatment job aides displayed in consultation room					
Yes	105 (49.3)	1		1	
No	5 (25.0)	1.52 (1.14 2.02)	0.004	0.64 (0.4 1.03)	0.07
Laboratory functionality in last 6 months					
All days of week	75 (56.8)	1		1	
Only on week days	38 (40.4)	0.71 (0.53 0.95)	0.02	0.47 (0.35 0.63)	0.000
Rarely open	9 (60.0)	1.06 (0.68 1.63)	0.08	0.63 (0.41 0.97)	0.037
Facility supervised in last 6 months					
Supervised by DHT	91 (46.1)	1			
Supervised by HSD	14 (82.6)	1.76 (1.34 2.29)	0.000	–	
Supervised by MoH	6 (75.0)	1.6 (1.04 2.45)	0.031	–	
Facility had anti-malarial stock-out in last 3 months					
Yes	30 (41.7)	1		1	
No	92 (54.4)	1.31 (1.06 1.77)	0.0000	1.17 (0.79 1.72)	0.43
Age of patient					
<5years	43 (49.3)	1		1	
≥5years	79 (51.3)	1.03 (0.798345 1.34)	0.08	1.07 (0.83 1.39)	0.543

### Diagnosis

The commonest malaria diagnostic technique is malaria RDT (90.87 %).

- “*We have RDTs, microscopy but we always use RDTs here because of the ease of use.”*-(Key informant)
- “*Malaria RDT have been very helpful, they have saved us from having stock*-*outs of anti*-*malarials; previously before RDT, in most cases we were treating malaria based on clinical features yet there are other problems which present with same symptoms and signs. It is not common to have stock*-*out these days because we give anti*-*malarial to patients following the laboratory investigations.*”-(Key informant) (Table 4).

**Table 4:** Laboratory diagnostic services

Variable	Frequency (N)	Percentage (%)
Common diagnostic method		
Malaria RDT	219	90.87
Microscopy	20	8.3
Both microscopy and malaria RDT	2	0.83
Laboratory diagnostic method used		
Malaria RDT	219	90.87
Both microscopy and malaria RDT	1	0.41
Do not know (not indicated)	21	8.71

### Prescription pattern

Of the 128 patients who had malaria confirmed present, 55 (42.97 %) were treated with an appropriate ACT. Seventy-two (56.25 %) of the same category of people were prescribed both an anti-malarial and at least an antibiotic. Prescription of non-ACT anti-malarials was low at only 3.5 % of all those prescribed anti-malarials. Out of the 88 patients who were parasitologically established to be malaria negative, 21 (23.86 %) were prescribed anti-malarial drugs. Nearly all those who presented with fever but had no laboratory tests done were prescribed anti-malarial drugs and/or antibiotics.

Healthcare workers said non-reliability of laboratories (25.27 %), delay in producing laboratory results (12.37 %), presence of cardinal malaria signs (26.34 %), presence of a “typical” clinical picture of malaria (8.06 %), and presence of signs and symptoms of malaria with no suspicion of any other differential diagnosis (27.96 %) being the drivers for not basing malaria management according to laboratory findings (Table 5).

**Table 5:** Reasons some health workers gave for not basing prescription on laboratory results

Variable	Frequency (N)	Percentage (%)
Laboratory not reliable	47	25.27
Delay in producing laboratory results	23	12.37
Presence of cardinal malaria signs	49	26.34
Presence of a “typical” clinical picture of malaria	15	8.06
Presence of signs and symptoms of malaria with no suspicion of any other differential diagnosis	52	27.96

## Discussion

The study aimed to assess clinician, health system and patient factors affecting adherence to guidelines in management of malaria among public healthcare workers. The results provide some explanations to help understand where possible interventions could improve the level of adherence to national guidelines. Adherence to the national guidelines is quite low at just above 50.6 %. Despite availability of clear guidelines and supervision, health workers do not follow recommended guidelines because of health system and health worker-related reasons. The factors associated with adherence to guidelines were: level of facility, cadre of health worker, presence of job aides in consultation rooms, recent attendance of a CME session on malaria and presence of a functional laboratory.

Better adherence to guidelines was more common practice at lower-level facilities. Related to this was the finding that lower-level cadres, such as nurses and nurse aides, adhered to guidelines better than higher-level cadres, such as clinical officers. This finding was similar to that observed in many other African countries where ACT was introduced [11–15, 17–19, 21, 23]. A study in Kenya by Zurovac et al. showed that nursing aides complied more closely to treatment guidelines than clinical officers [15]. Previous Ugandan studies have shown similar findings with most showing better adherence by clinical officers and nurses than doctors, who are considered better trained [16, 17, 21]. Possible explanations for this could be that higher level trained workers such as medical officers and clinical officers believe they have higher medical acumen and hence a tendency not to use the guidelines. Indeed, many of the clinical officers interviewed reported that their reasons for not adhering to these guidelines were because either the laboratory was not reliable or they could rely on presence of “typical” malaria signs to make a decision.

It could also be that higher-level cadres tend to be in administrative positions and are often missed out for supervision and training. This could be reflective of the pre-service training gaps that include non-alignment of medical school curriculum to national standards.

Unlike most of the previous studies in Uganda and the rest of Africa, this study found presence of job aides and recent participation in a facility-based medical education session was associated with improved adherence to guidelines. This could be explained by there being an ongoing project supporting the district to improve its capacity to manage malaria. As part of the project, in-facility education sessions were conducted and RDTs supplied. It will be interesting to re-assess this after the end of the project.

Thirdly, awareness by clinicians that there was a functional laboratory on most days was associated with better adherence to guidelines. This has not been explored by most of the other studies on this subject [11–19]. Many health workers who did not adhere to guidelines reported that this was because of the delay in getting results from the laboratory. It appears that the key factor in improving diagnostics for malaria was the introduction of RDTs as the vast majority of tests were done with RDTs. Others have shown the availability of RDTs improved malaria case management [21, 23, 24]. Widespread provision of malaria RDTs could well play a significant role in reducing the persistent problem of malaria overdiagnosis and contribute to reduced risk of malaria under-treatment. This is more so in a setting in the most affected parts of the world where microscopic diagnosis is hampered by unavailability of microscopes and electricity.

An important key finding was that prescription of ACT when a malaria diagnosis was made was almost universal with only about 5 % not prescribing ACT. This is a much higher rate than that seen in previous Ugandan studies [18, 19, 21, 23, 25]. This could have been affected by local factors such as limited ACT stocks, ongoing malaria project in the district, and the fact that this was limited to the public sector. Private facilities have been known to be more likely to prescribe non-ACT. This study was limited to the public sector unlike other studies that have included some private sector facilities [24, 25].

This study highlights the significant shortage of human resources in rural districts. More than 60 % of the patients were attended to by nurses and nursing aides. This, despite the fact that nursing aides are not expected to be managing patients according to current health guidelines in Uganda [20].

The lack of association of age of health worker, duration of employment, supervision, educational level and age of patient, in-service training is consistent with the majority of other studies on this subject [11–19, 22, 23, 26].

## Limitations

This study had a number of limitations. First, the study was conducted in public health facilities; this limits generalizability to these facilities. Second, it was conducted in one district in which there was an ongoing project addressing malaria management. This might not be replicated in other districts without such support. Third, despite targeting clients at exit, the study results might have been subject to bias from the Hawthorne effect, since the health workers were aware that their facilities and actions were being observed. The study did not assess impact of patient demands and the study was conducted over a short period of time. Additionally HC IIs were not studied yet they do manage malaria cases. This limits generalizability of findings to all levels of the healthcare system.

## Conclusions

This study found that health worker case management decisions were associated with health system and health worker-related factors that are potentially modifiable. Although some recent evidence suggests that use of text message reminders to health workers might improve provider behaviours at low cost, evidence-based interventions to improve rural health worker adherence to management guidelines are generally lacking or show mixed results [26–28]. This study strengthens the case for provision of aides, targeted, facility-based, continuing medical education on malaria, and targeting higher level, trained, health cadres. Another key issue is the need to ensure laboratories or at the very least RDT testing services are readily available to reduce waste of resources due to over-diagnosis [29–31]. Given the lack of good evidence on what works, interventional studies to evaluate the impact of these approaches is required to provide definitive guidance on what interventions best translate into better malaria treatment practices in Uganda.

## Figures and Tables

**Fig. 1:**
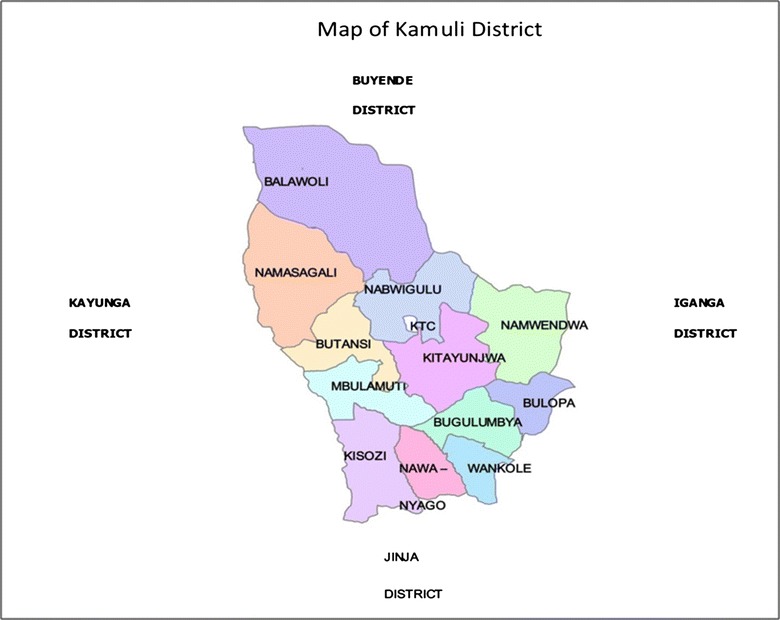
Map showing Kamuli District. It lies at an average altitude of 1083 m above sea level and extends 00–56′ North/330–05′ East up to 010–20′ North/330–15′ East. It has a total land area of 1622 km^2^ and 62.64 km^2^ (3.9 %) of water. The District is among the most underdeveloped and poor in Uganda (Ministry of finance, planning and economic development (MOFPED), 2003)

## Appendix 1

See Table 6.

**Table 6:** Health Centers and Health worker sampling frame: Population of Kamuli Health Care workers per station

No	Institution	Medical officers	Clinical officers	Nursing officers	Enrolled nurses	Enrolled midwives	Nursing assistant	Total
1	DHO office	1	0	1	0	0	0	2
2	Kamuli General Hospital	4	9	20	25	18	15	91
3*	Balawoli HC III	0	2	1	2	3	1	9
4*	Namasagali HC III	0	2	2	2	3	2	11
5*	Nabirumba HC III	0	2	1	2	3	1	9
6	Namaira HC II	0	0	0	0	1	1	2
7	Kibuye HC II	0	0	1	0	1	0	2
8	Kiige HC II	0	0	0	2	0	0	2
9	Kawaga HC II	0	0	0	1	0	0	1
10	Kasolwe HC II	0	0	0	0	3	0	3
11	Kamuli youth centre HC II		0	0	1	1	0	2
12	Nawankofu HC II	0	0	0	0	1	1	2
13	Kagumba HC II	0	0	0	0	1	1	2
14	Namunyingi HC II	0	0	0	1	2	1	4
15*	Nankandulo HC IV	1	4	3	5	3	2	18
16*	Mbulamuti HC III	0	3	1	4	2	3	13
17*	Lulyambuzi HC III	0	2	1	2	4	1	10
18*	Bupadhengo HC III	0	2	1	2	2	2	9
19*	Bugulumbya HC III	0	3	3	1	2	1	10
20	Buluya HC II	0	0	1	0	1	1	3
21	Kiyunga HC II	0	0	0	1	0	1	2
22	Nawandyo HC II	0	0	0	1	1	1	3
23	Kasambira HC II	0	0	1	0	0	1	2
24	Nawantumbi HC II	0	0	1	0	1	1	3
25	Buwoya HC II	0	0	0	0	1	1	2
26	Bubago HC II	0	0	0	1	1	0	2
27	Luzinga HC II	0	0	0	0	0	1	1
28	Kiyunga-Bukakanda HC II		0	1	0	0	0	1
29*	Namwendwa HC IV	2	3	3	3	3	2	16
30*	Butansi HC III	0	2	1	2	3	1	9
31*	Kitayunjwa HC III	0	2	3	2	2	2	11
32*	Bulopa HC III	0	2	1	2	3	1	9
33	Kinu HC II	0	0	0	1	1	0	2
34	Kyeeya HC II	0	0	0	1	1	0	2
35	Nabirama HC II	0	0	1	1	0	1	3
36	Kinawampere HC II	0	0	0	1	0	1	2
37	Busota HC II	0	0	0	0	1	1	2
	Total	8	38	48	66	69	48	277

* Facilities sampled

## Appendix 2

See Fig. 1.
